# Distortion-Free Magnetic Tracking of Metal Instruments in Image-Guided Interventions

**DOI:** 10.3390/s24165364

**Published:** 2024-08-20

**Authors:** Eoin Higgins, Daragh Crowley, Christian van den Bosch, Pádraig Cantillon-Murphy

**Affiliations:** 1 Tyndall National Institute, Lee Maltings, Dyke Parade, T12 R5CP Cork, Ireland; daragh.crowley@tyndall.ie (D.C.); p.cantillonmurphy@ucc.ie (P.C.-M.); 2 School of Engineering, University College Cork, College Road, T12 K8AF Cork, Ireland

**Keywords:** electromagnetic tracking, surgical navigation, inductive sensor, magnetic modelling

## Abstract

Electromagnetic tracking (EMT) can benefit image-guided interventions in cases where line of sight is unavailable. However, EMT can suffer from electromagnetic distortion in the presence of metal instruments. Metal instruments are widely used in laparoscopic surgery, ENT surgery, arthroscopy and many other clinical applications. In this work, we investigate the feasibility of tracking such metal instruments by placing the inductive sensor within the instrument shaft. We propose a magnetostatic model of the field within the instrument, and verify the results experimentally for frequencies from 6 kHz to 60 kHz. The impact of the instrument’s dimensions, conductivity and transmitting field frequency is quantified for ranges representative of typical metal instruments used in image-guided interventions. We then performed tracking using the open-source Anser EMT system and quantify the error caused by the presence of the rod as a function of the frequency of the eight emitting coils for the system. The work clearly demonstrates why smaller tool diameters (less than 8 mm) are less susceptible to distortion, as well as identifying optimal frequencies (1 kHz to 2 kHz) for transmitter design to minimise for distortion in larger instruments.

## 1. Introduction

When line of sight is unavailable, electromagnetic tracking (EMT) is the gold standard for surgical navigation. It has seen use across numerous clinical applications such as bronchoscopy [1,2,3], orthopaedic surgery [4] and cardiovascular surgery [5]. Furthermore, a number of commercial EMT systems are available, including the NDI Aurora and Trakstar (Northern Digital Inc., Waterloo, ON, Canada) and the Polhemus Fastrak (Polhemus Inc., Colchester, VT, USA). In ideal environments, these systems can routinely achieve sub-millimetre tracking accuracy [6]. Difficulties arise in applications which necessitate the tracking of metallic instruments [7,8]. These procedures include needle tracking [9], the tracking of ultrasound probes [10,11] and orthopaedic procedures [12,13]. Such instruments can cause distortion of the magnetic field which is used for sensor localisation, resulting in larger tracking errors. A number of approaches have been proposed to minimise the impact of magnetic field distortion in metallic instrument tracking. One possibility is to offset the electromagnetic sensor from the instrument shaft [14]. However, this can lead to amplified tracking errors in orientation estimates [15] and increased instrument diameters. Another possibility is the fusion between EMT and optical tracking [16]; however, the resulting system is still susceptible to visual occlusion. In this paper, we investigate the feasibility of performing EMT with a sensor placed within the shaft of metallic surgical instruments. To this end, we investigate the shielding effect of a nonmagnetic conductive rod using analytical and experimental approaches. The tracking accuracy in the presence of metallic surgical instruments is also quantified.

## 2. Mathematical Formalism

Electromagnetic fields are governed by Maxwell’s equations which, in the case of linear, isotropic and nonmagnetic media, are given by [17]

(1) $$\begin{array}{cc} \nabla \times E & =-\frac{\partial B}{\partial t} \end{array}$$

(2) $$\begin{array}{cc} \nabla \times B & =\frac{\partial \varepsilon_{0}E}{\partial t}+\mu_{0}J \end{array}$$

(3) $$\begin{array}{cc} \nabla \cdot B & =0 \end{array}$$

where B is the magnetic flux density, E is the electric field, J is the conduction current density and $\varepsilon_{0}$ and $\mu_{0}$ are, respectively, the electrical permittivity and magnetic permeability of free space. However, in electromagnetic tracking, we are only interested in slowly oscillating magnetic fields. Therefore, in this work, we limit our analysis to quasistatic magnetic fields. In this regime, Ampere’s law reduces to

(4) $$\nabla \times B=\mu_{0}J$$

**Figure 1:**
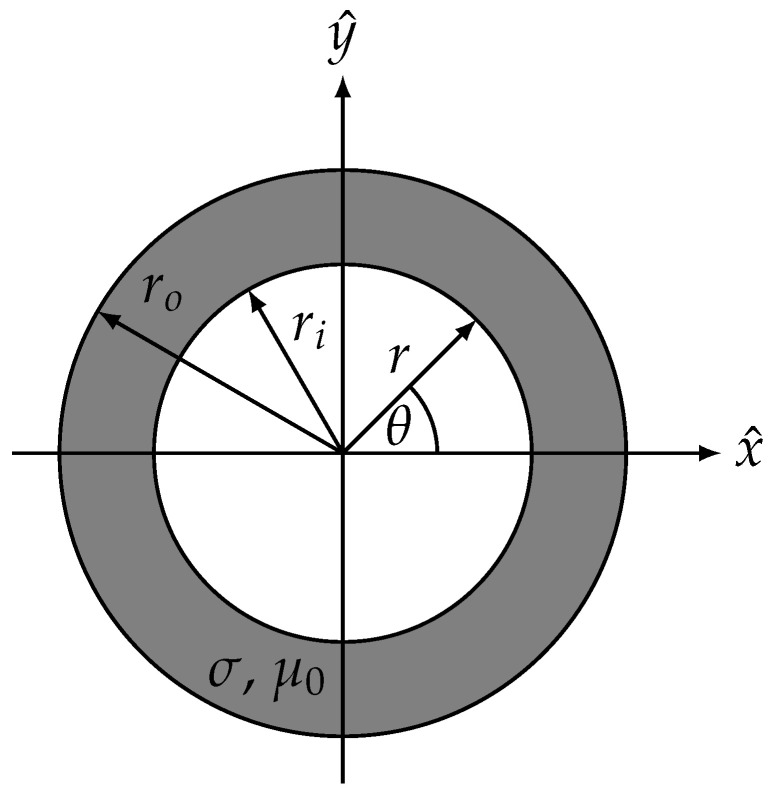
An infinitely long cylindrical rod with inner radius $r_{i}$ and outer radius $r_{o}$. The axis of the rod is oriented along the *z*-direction. The radial and azimuthal coordinates are denoted by *r* and θ.

To investigate the shielding effect of a surgical instrument, we approximate it as an infinite cylindrical rod with inner radius $r_{i}$, outer radius $r_{o}$ and conductivity σ. Such a cylindrical rod can be seen in Figure 1. We adopt the obvious choice of a cylindrical coordinate system with the rod axis oriented along the *z* axis. In light of (3), we introduce a magnetic vector potential A such that

(5) B=∇×A.

Next, we express Maxwell’s equations in terms of this magnetic vector potential. Equation (1) implies the existence of a scalar potential ψ such that

(6) $$E+\frac{\partial A}{\partial t}=\nabla \psi .$$

By combining (4) and (6) and utilising Ohm’s law J=σE, we have

(7) $$\nabla^{2}A-\nabla \left(\nabla \cdot A-\mu_{0}\sigma \psi \right)=\mu_{0}\sigma \frac{\partial A}{\partial t}.$$

In the case where the externally applied field is oscillating sinusoidally at frequency *f*, we can adopt a phasor convention such that $\frac{\partial A}{\partial t}=2\pi jfA$. The phasor transformation combined with a choice of gauge given by $\nabla \cdot A=\mu_{0}\sigma \psi$ reduces (7) to the vector Helmholtz equation

(8) $$\nabla^{2}A+k^{2}A=0$$

where $k=\sqrt{-2\pi jf\mu_{0}\sigma }$.

### 2.1. Conductive Rod in a Transverse Magnetic Field

In the case of the transverse field $B_{T}=B_{0}\hat{x}$, where $B_{0}$ is a time-dependent amplitude, the magnetic vector potential can be expressed as a single *z*-directed component $A_{T}$, given by

(9) $$A_{T}=A_{T}\hat{z}=B_{0}r\sin\left(\theta \right)\hat{z}$$

where *r* is the radial coordinate and θ is the azimuthal coordinate. In light of (9), we need only consider the *z* component of the Helmholtz equation. Therefore, we have $A=A_{z}\hat{z}$ and (8) reduces to the scalar Helmholtz equation. We can expand the Laplacian in cylindrical coordinates to give

(10) $$r^{2}\frac{\partial^{2}A_{z}}{\partial r^{2}}+r\frac{\partial A_{z}}{\partial r}+\frac{\partial^{2}A_{z}}{\partial \theta^{2}}+k^{2}r^{2}A_{z}=0$$

where the derivatives with respect to *z* are zero in the case of an infinite cylinder. The complete solution for $A_{z}$ in the three regions of the domain is given by [18]

(11) $$A_{z}=\sin\left(n\theta \right)\left\{\begin{array}{cc} a_{1}r^{n}+b_{1}r^{-n}, & r<r_{i}\\ a_{2}J_{n}\left(kr\right)+b_{2}Y_{n}\left(kr\right), & r_{i}\leq r\leq r_{o}\\ a_{3}r^{n}+b_{3}r^{-n}, & r>r_{o} \end{array}\right.$$

where $J_{n}$ and $Y_{n}$ are the Bessel functions of the first and second kind, respectively. The constants *n*, $a_{1,2,3}$ and $b_{1,2,3}$ are to be determined from boundary conditions. The first boundary condition we impose is the finiteness of the solution at the origin. This implies $b_{1}=0$. Next, we demand that as *r* tends to infinity, $A_{z}$ must approach the vector potential $A_{T}$ of the imposed external field. This gives n=1, and $a_{3}=B_{0}$.

Next, we impose the continuity of the normal component of the magnetic flux density B and continuity of the tangential component of the magnetic field strength H along each interface. This yields four conditions, namely

(12) $$\begin{array}{cc} a_{1}r_{i} & =a_{2}J_{1}\left(kr_{i}\right)+b_{2}Y_{1}\left(kr_{i}\right) \end{array}$$

(13) $$\begin{array}{cc} B_{0}r_{o}+\frac{b_{3}}{r_{o}} & =a_{2}J_{1}\left(kr_{o}\right)+b_{2}Y_{1}\left(kr_{o}\right) \end{array}$$

(14) $$\begin{array}{cc} a_{1} & =a_{2}J_{1}^{\prime }\left(kr_{i}\right)+b_{2}Y_{1}^{\prime }\left(kr_{i}\right) \end{array}$$

(15) $$\begin{array}{cc} B_{0}+\frac{b_{3}}{r_{o}^{2}} & =a_{2}J_{1}^{\prime }\left(kr_{o}\right)+b_{2}Y_{1}^{\prime }\left(kr_{o}\right) \end{array}$$

where the primes denote derivatives. The solutions to this system of equations are

(16) $$\begin{array}{cc} a_{1} & =\frac{4B_{0}}{k^{2}\pi r_{i}^{2}\left(J_{2}\left(kr_{i}\right)Y_{0}\left(kr_{o}\right)-Y_{2}\left(kr_{i}\right)J_{0}\left(kr_{o}\right)\right)} \end{array}$$

(17) $$\begin{array}{cc} a_{2} & =-\frac{2B_{0}Y_{2}\left(kr_{i}\right)}{kJ_{2}\left(kr_{i}\right)Y_{0}\left(kr_{o}\right)-kY_{2}\left(kr_{i}\right)J_{0}\left(kr_{o}\right)} \end{array}$$

(18) $$\begin{array}{cc} b_{2} & =\frac{2B_{0}J_{2}\left(kr_{i}\right)}{kJ_{2}\left(kr_{i}\right)Y_{0}\left(kr_{o}\right)-kY_{2}\left(kr_{i}\right)J_{0}\left(kr_{o}\right)} \end{array}$$

(19) $$\begin{array}{cc} b_{3} & =\frac{B_{0}r_{o}^{2}\left(J_{2}\left(kr_{i}\right)Y_{2}\left(kr_{o}\right)-Y_{2}\left(kr_{i}\right)J_{2}\left(kr_{o}\right)\right)}{J_{2}\left(kr_{i}\right)Y_{0}\left(kr_{o}\right)-Y_{2}\left(kr_{i}\right)J_{0}\left(kr_{o}\right)} \end{array}$$

**Figure 2:**
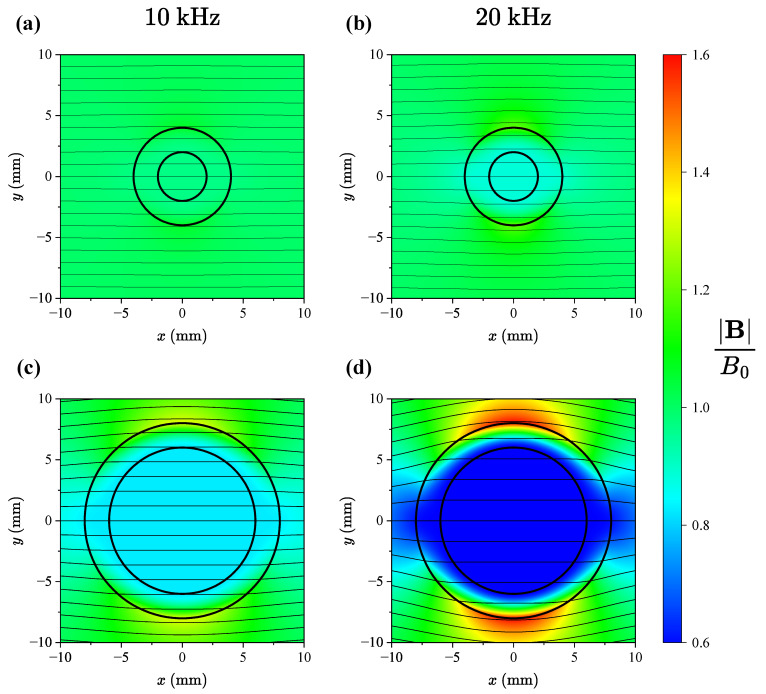
The normalised magnitude of the magnetic flux density for two different excitation frequencies for a conductivity of σ=1.35 MS/m. In subfigures (**a**) and (**b**), we have $r_{i}=$ 2 mm and $r_{o}=$ 4 mm, while in subfigures (**c**) and (**d**), we have $r_{i}=$ 6 mm and $r_{o}=$ 8 mm.

By using Equation (5), we can obtain the complete solution for the magnetic flux density in each region of the domain. The normalised magnitude of the magnetic flux density can be seen in Figure 2. We have evaluated the solution for two different rod geometries for two different frequencies of the externally applied field. As we are primarily interested in the magnetic flux density within the rod, we now turn our attention to the solution in the innermost region. For $r<r_{i}$, we have

(20) $$B=\frac{1}{r}\frac{\partial A_{z}}{\partial \theta }\hat{r}-\frac{\partial A_{z}}{\partial r}\hat{\theta }=a_{1}\hat{x}=\frac{4B_{0}\hat{x}}{k^{2}\pi r_{i}^{2}\left(J_{2}\left(kr_{i}\right)Y_{0}\left(kr_{o}\right)-Y_{2}\left(kr_{i}\right)J_{0}\left(kr_{o}\right)\right)}.$$

### 2.2. Conductive Rod in a Longitudinal Magnetic Field

If the rod is placed in a longitudinal field given by $B_{L}=B_{0}\hat{z}$, we can express the magnetic vector potential as

(21) $$A_{L}=A_{L}\hat{\theta }=\frac{1}{2}B_{0}r\hat{\theta }$$

With this in mind, we need only consider the θ component of the Helmholtz equation. Therefore, we have $A=A_{\theta }\hat{\theta }$. We can expand the azimuthal component of the vector Laplacian in cylindrical coordinates to give

(22) $$r^{2}\frac{d^{2}A_{\theta }}{dr^{2}}+r\frac{dA_{\theta }}{dr}+\left(k^{2}r^{2}-1\right)A_{\theta }=0$$

where the derivative with respect to *z* and θ are zero.

The complete solution for $A_{\theta }$ in all regions is therefore given by

(23) $$A_{\theta }=\left\{\begin{array}{cc} \frac{c_{1}}{r}+d_{1}r, & r<r_{i}\\ c_{2}J_{1}\left(kr\right)+d_{2}Y_{1}\left(kr\right), & r_{i}\leq r\leq r_{o}\\ \frac{c_{3}}{r}+d_{3}r, & r>r_{o} \end{array}\right.$$

The constants $c_{1,2,3}$ and $d_{1,2,3}$ are to be determined from boundary conditions. We again impose the finiteness of the solution at the origin. This implies $c_{1}=0$. Next, we demand that as *r* tends to infinity, $A_{\theta }$ must approach the vector potential $A_{L}$ of the imposed external field. This yields $d_{3}=B_{0}/2$.

Next, we impose the continuity of $A_{\theta }$ and $B_{z}$ at the metal air interfaces. This gives four equations:

(24) $$\begin{array}{cc} d_{1}r_{i} & =c_{2}J_{1}\left(kr_{i}\right)+d_{2}Y_{1}\left(kr_{i}\right) \end{array}$$

(25) $$\begin{array}{cc} \frac{c_{3}}{r_{o}}+\frac{1}{2}B_{0}r_{o} & =c_{2}J_{1}\left(kr_{o}\right)+d_{2}Y_{1}\left(kr_{o}\right) \end{array}$$

(26) $$\begin{array}{cc} 2d_{1} & =c_{2}kJ_{0}\left(kr_{i}\right)+d_{2}kY_{0}\left(kr_{i}\right) \end{array}$$

(27) $$\begin{array}{cc} B_{0} & =c_{2}kJ_{0}\left(kr_{o}\right)+b_{2}kY_{0}\left(kr_{o}\right). \end{array}$$

The solution to this system of equations is

(28) $$\begin{array}{cc} a_{1} & =\frac{4B_{0}}{k^{2}\pi r_{i}^{2}\left(J_{2}\left(kr_{i}\right)Y_{0}\left(kr_{o}\right)-Y_{2}\left(kr_{i}\right)J_{0}\left(kr_{o}\right)\right)} \end{array}$$

(29) $$\begin{array}{cc} a_{2} & =-\frac{B_{0}Y_{2}\left(kr_{i}\right)}{kJ_{2}\left(kr_{i}\right)Y_{0}\left(kr_{o}\right)-kY_{2}\left(kr_{i}\right)J_{0}\left(kr_{o}\right)} \end{array}$$

(30) $$\begin{array}{cc} b_{2} & =\frac{B_{0}J_{2}\left(kr_{i}\right)}{kJ_{2}\left(kr_{i}\right)Y_{0}\left(kr_{o}\right)-kY_{2}\left(kr_{i}\right)J_{0}\left(kr_{o}\right)} \end{array}$$

(31) $$\begin{array}{cc} b_{3} & =\frac{B_{0}r_{o}^{2}\left(J_{2}\left(kr_{i}\right)Y_{2}\left(kr_{o}\right)-Y_{2}\left(kr_{i}\right)J_{2}\left(kr_{o}\right)\right)}{2J_{2}\left(kr_{i}\right)Y_{0}\left(kr_{o}\right)-2Y_{2}\left(kr_{i}\right)J_{0}\left(kr_{o}\right)} \end{array}$$

The magnetic flux density in the innermost region, where $r<r_{i}$, is given by

(32) $$B=\left(\frac{A_{\theta }}{r}-\frac{dA_{\theta }}{dr}\right)\hat{z}=2d_{1}\hat{z}=\frac{4B_{0}\hat{z}}{k^{2}\pi r_{i}^{2}\left(J_{2}\left(kr_{i}\right)Y_{0}\left(kr_{o}\right)-Y_{2}\left(kr_{i}\right)J_{0}\left(kr_{o}\right)\right)}.$$

### 2.3. Magnetic Field Solutions in the Innermost Region

In the case of a nonmagnetic conducting rod, the solution for the magnetic field in the innermost region is the same for both transverse and longitudinal fields. Therefore, we conclude that the effect of the rod on a spatially uniform field is independent of orientation.

**Figure 3:**
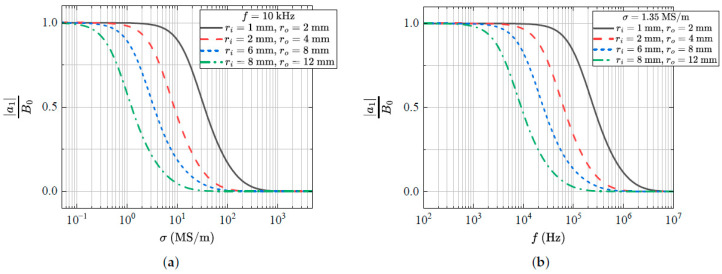
The magnitude of the normalised magnetic flux density as a function of (**a**) conductivity and (**b**) frequency for a number of different rod geometries.

**Figure 4:**
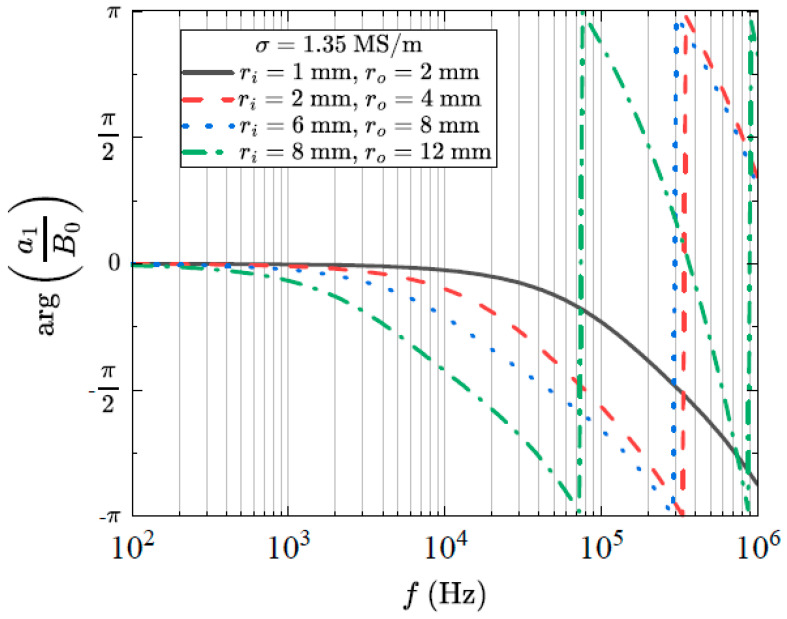
The phase difference between the magnetic flux density inside and outside the rod as a function of frequency for a number of different rod geometries.

The field inside the rod is in the same direction as the external field; however, it has a magnitude given by $\left|a_{1}\right|$ and a phase difference given by $\arg(a_{1})$. The normalised field magnitude as a function of frequency and conductivity for a number of rod geometries can be seen in Figure 3. In (a), we have fixed the frequency at f=10 kHz and varied the conductivity. In (b), we have fixed the conductivity at σ=1.35 MS/m and varied the frequency. This is a typical value for the conductivity of medical-grade stainless steel. We note that the shielding effect is strongly dependent on geometry, frequency and conductivity. The phase difference induced by the rod can be seen in Figure 4. Here, we have again fixed the conductivity at σ=1.35 MS/m and varied the frequency.

## 3. Methods

In order to verify the analytical solutions introduced in Section 2, we conducted a number of experiments to measure the shielding effect of stainless steel rods. The details of each rod can be seen in Table 1. In contrast to the magnetic field used for magnetic tracking, the magnetic field used in these experiments is spatially uniform. These experiments yield the range of magnetic field frequencies and corresponding instrument diameters, for which the inductive shielding effects are acceptable for tracking.

### 3.1. Helmholtz Coil Driver Design

**Figure 5:**
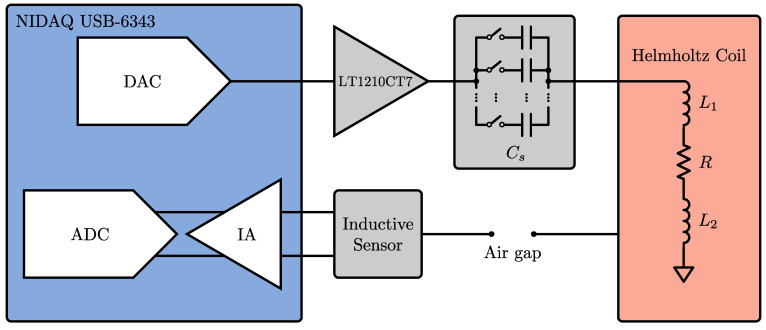
A schematic of the circuit used to drive the Helmholtz coils.

To quantify this effect, a spatially uniform, transverse magnetic field of known frequency was used. A convenient method for producing a uniform field is a Helmholtz coil. This consists of two identical co-axial coils which are connected in series. The result is a spatially uniform magnetic field in the cylindrical region oriented along the axis of the coils. This work employed a 300 mm diameter Helmholtz coil (Ferronato BH300HF-3-B, Serviciencia S.L.U, Málaga, Spain). Each coil consists of eight turns with the pair of coils having a self-inductance of 93 µH and a field-to-current ratio of 54.2 µT/A. To increase the current that can be driven in the coils, a series capacitor is used to tune the resonant frequency of the circuit. Assuming a simple lumped model for the Helmholtz coil in series with the capacitor, the self-resonant frequency $f_{0}$ is given by

(33) $$f_{0}=\frac{1}{2\pi \sqrt{C_{s}\left(L_{1}+L_{2}\right)}}$$

where $L_{1}$ and $L_{2}$ are the self-inductances of each of the coils, respectively, and $C_{s}$ is the chosen series capacitance. A schematic of the circuit used to drive the Helmholtz coil can be seen in Figure 5. A National Instruments USB-6343 data acquisition unit is used to drive the sinusoidal signal, which is then amplified using an LT1210CT7 power amplifier. A relay-switched capacitor bank is used to select the value of the series capacitor. The capacitance is chosen such that the circuit is driven as close as possible to resonance. The generated field was verified using a calibrated triaxial magnetic field probe (ELT-400, Narda Safety Test Solutions GmbH, Pfullingen, Germany).

### 3.2. Influence of Transmitter Frequency

**Figure 6:**
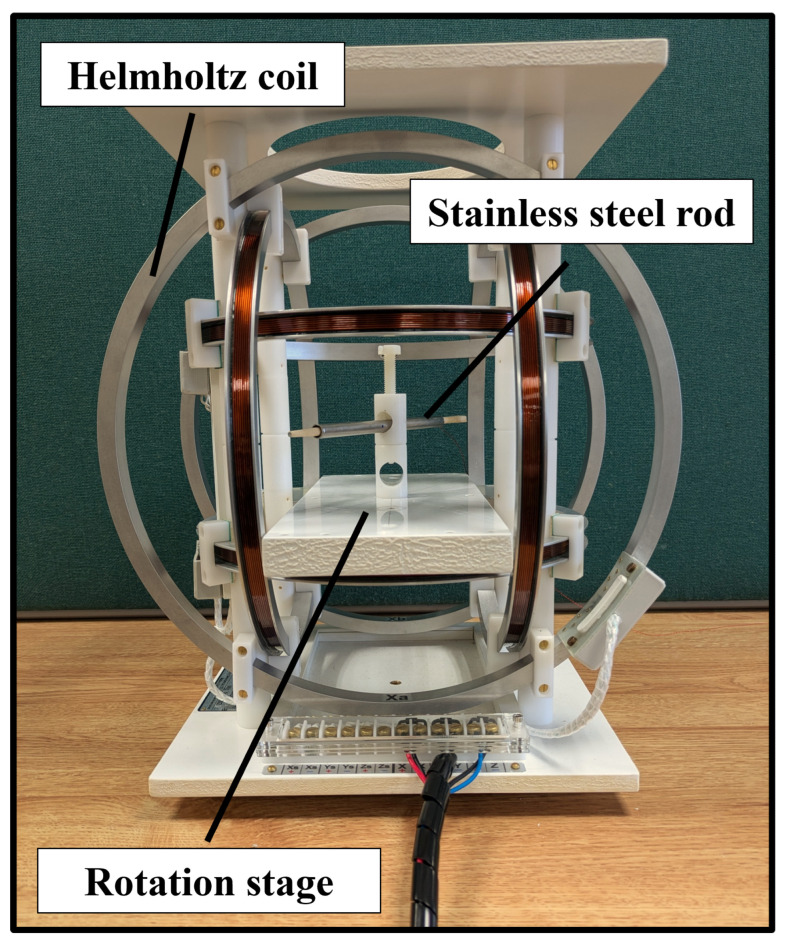
The experimental setup used to measure the shielding effect and phase induced by stainless steel rods.

Using the Helmholtz coil, a homogeneous field was then driven at a given frequency *f*. The inductive sensor was aligned parallel to the magnetic field and, without any metallic rods present, the voltage $v_{0}$ induced across the sensor was measured. Measurements were taken over an interval of twenty seconds at a sampling rate of 250 kS/s. This voltage was validated to be linear in both frequency and applied magnetic field strength for the region of interest. Next, the inductive sensor was fixed axially within the centre of each stainless steel rod. The rod was placed into the Helmholtz coil with an angle α between the sensor axis and the driven magnetic field. The corresponding voltage $v_{i}$ was measured. This was repeated for frequencies from 4 kHz to 60 kHz and angles α of $0^{\circ }$, $30^{\circ }$ and $45^{\circ }$. The experimental setup can be seen in Figure 6. To determine the shielding effect of the rod, we are interested in the ratio $\left|B_{i}/B_{0}\right|$, where $B_{i}$ is the amplitude of the magnetic flux density within rod *i* and $B_{0}$ is the amplitude of the magnetic flux density during the control measurement when no rod is present. To obtain the magnitude of the field, we take the FFT of the the measured voltage signal and extract the amplitude corresponding to the frequency of the driven magnetic field.

A similar procedure was followed to measure the phase difference induced by the presence of the rods. First, using the FFT and with no rod present, we calculated the phase difference $\phi_{0}$ between the voltage across the inductive sensor and the current through the Helmholtz coils. In the same way, we obtained the phase difference $\phi_{i}$ between the voltage across the inductive sensor and the current through the Helmholtz coil in the presence of rod *i*. We were then interested in the additional phase difference $\Delta \phi_{i}$ induced by the presence of the rod. This is given by

(34) $$\Delta \phi_{i}=\phi_{i}-\phi_{0}.$$

This was repeated for a range of frequencies from 4 kHz to 60 kHz.

### 3.3. Impact on Electromagnetic Tracking

To assess the feasibility of tracking with a sensor within metallic surgical instruments, we perform grid measurements with an inductive sensor placed within a stainless steel rod. This work employed the open-source Anser EMT tracking system [19]. The Anser EMT system uses a field generator with eight coils to create a spatially unique and time-varying electromagnetic field. Frequency division multiplexing is used with each emitter coil driven at a different frequency.

**Figure 7:**
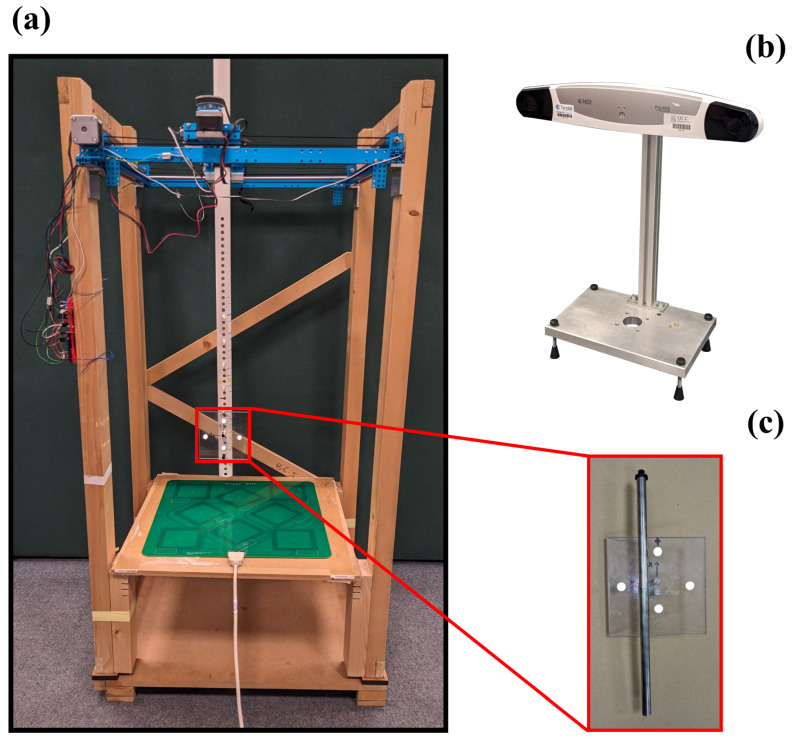
The experimental setup with (**a**) the robotic positioning system, (**b**) the optical tracking system and (**c**) the optical tool with IR markers and the stainless steel rod and sensor rigidly attached.

The Anser EMT tracking system was used to track an inductive sensor with a diameter of 0.45 mm and a length of 8.2 mm (NDI 610158, Northern Digital Inc., Waterloo, ON, Canada). An optical tracking system (NDI Polaris, Northern Digital Inc., Waterloo, ON, Canada) was used as the ground truth. The experimental setup can be seen in Figure 7.

To measure the accuracy over the volume of interest, a robot was used to move the sensor in a grid at a fixed height above the field generator. Each grid comprised N= 100 points organised in a 10×10 grid, occupying an area of 25 cm × 25 cm in the xy plane. At each grid location, EMT and optical measurements were recorded simultaneously. With an inductive sensor fixed co-axially within each rod, grid measurements were taken at heights of 210 mm and 270 mm above the field generator. Grid measurements were repeated for two sensor orientations; *z*- and *y*-directed. A full grid measurement was performed with the emitter coils driven at different frequencies from 1 kHz to 20 kHz.

Once measurements were completed, Horn’s absolute orientation algorithm [20] was used to align the optical and EMT reference frames. The error between these aligned grids was then defined as our tracking error.

## 4. Results and Discussion

### 4.1. The Influence of Transmitter Frequency

**Figure 8:**
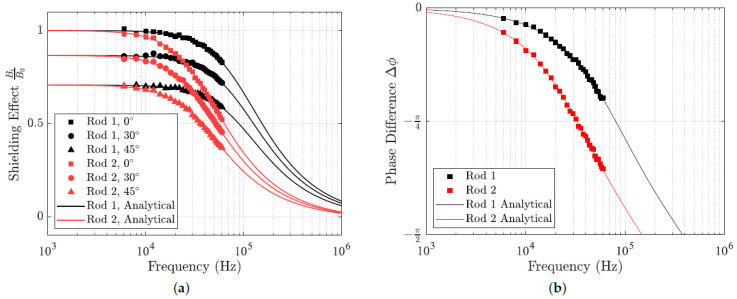
Experimental measurements of (**a**) the shielding effect and (**b**) induced phase for the two stainless steel rods. Analytical results given by (32) are denoted by solid curves.

The experimental measurements of the shielding effect and induced phase difference can be seen in Figure 8. The solid curves denote the theoretical curves computed using (32) with the rod parameters from Table 1. In both cases, there is good agreement between the measured results and the expected analytical curves. As expected, in both cases, the rod with larger diameter has a greater effect on the magnetic field. For the range of frequencies employed in electromagnetic tracking, the magnetostatic approximation of Maxwell’s equations accurately predicts for the effect of metal instruments. Relative to a uniform external field, the magnetic field within a cylindrical instrument has an attenuated magnitude and an additional induced phase difference.

### 4.2. Impact on Electromagnetic Tracking

Calibration of the electromagnetic sensor was performed by collecting EMT and optical measurements in two orientations at a height of 250 mm above the field generator. These measurements were completed without the presence of any distorters. Using a subset of 50 points from this grid, a calibration was performed to ensure accurate tracking in the region of interest. The remaining points were used to measure the accuracy of the Anser EMT system at each frequency.

The same calibration was used when measuring the tracking performance in the presence of each stainless steel rod for N= 100 points across each *xy* plane. The Euclidean error $e_{i}$ at each grid point *i* is given by

(35) $$e_{i}=\sqrt{\Delta x^{2}+\Delta y^{2}+\Delta z^{2}}$$

where Δx, Δy and Δz are the deviations between the aligned electromagnetic and optical positions. The root-mean-squared error RMSE is then given by

(36) $$\mathrm{RMSE}=\sqrt{\frac{1}{N}\sum_{i=1}^{N}e_{i}^{2}},$$

and the mean error ME is given by

(37) $$\mathrm{ME}=\frac{1}{N}\sum_{i=1}^{N}e_{i}.$$

The standard deviation of the errors is given by

(38) $$\mathrm{STD}=\sqrt{\frac{N}{N-1}\left({\mathrm{RMSE}}^{2}-{\mathrm{ME}}^{2}\right)}.$$

**Figure 9:**
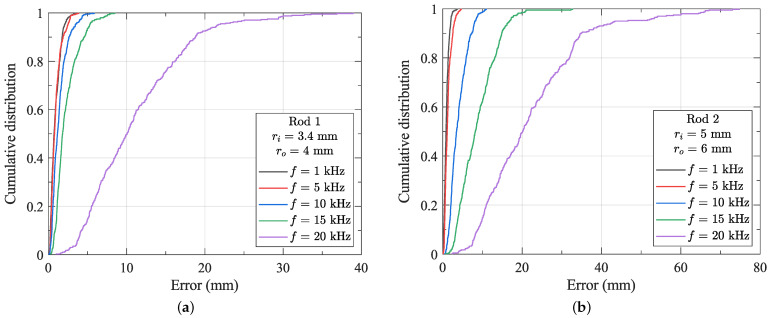
The cumulative distribution of the tracking errors with a sensor fixed within (**a**) rod 1 and (**b**) rod 2.

The results for each rod and each frequency can be seen in Table 2. The cumulative distribution of the tracking error as a function of emitter coil frequency with the sensor fixed within each rod can be seen in Figure 9. The optical system has a reported volumetric accuracy of 0.15 mm RMSE [21]. The tracking error shows a strong dependency on frequency, with much larger errors at increasing frequency. From these results, it is clear that for the distortionless tracking of metal instruments of 8 mm or greater, the frequency of the transmitter magnetic field must be less than 1 kHz. However, such low-frequency tracking may not provide sufficient update rates for some applications. To enable tracking at higher frequencies, compensation for the effects of the instrument is required. In some applications, tracking errors may be acceptable at 5 kHz or 10 kHz. Alternatively, smaller instruments (<8 mm outer diameter) can be accurately tracked at significantly higher frequencies and update rates. Finally, the impact of dynamic field effects (e.g., instrument motion) has not been considered in this work. However, given that such motion in clinical applications is typically highly controlled and limited, it can be reasonably expected that the quasistatic effects outlined in this paper will dominate the distortion of the magnetic field.

## 5. Conclusions

In this work, we investigate the feasibility of tracking metal instruments typical of image-guided interventions using electromagnetic tracking with an inductive sensor placed within the instrument shaft. Analytical models of the instrument as an infinite conductive rod investigated the effect of this distorter on the magnetic field within the shaft. The proposed model neglects end-effects introduced by an instrument of finite extent and the external field was assumed to be spatially uniform. However, in the case of the electromagnetic tracking, neither of these criteria are typically encountered. To enable tracking, there must be a gradient present in the generated magnetic field, and hence, the field is not spatially uniform. The rod is found to have an effect which is strongly dependent on the tracking frequency, material properties and the rod geometry. We experimentally verified these results with a homogeneous field.

The impact of distortion was assessed for larger-diameter rods using an inductive sensor within the shaft and the open-source Anser EMT system. For typical conductivities in steel rods with outer diameters less than 8 mm, it was demonstrated that accurate tracking is possible up to around 5 kHz. For small-enough instrument diameters (<8 mm), the RMSE of less than 2 mm at 10 kHz may be sufficiently low that it can be neglected in comparison to image-to-patient registration errors. These registration errors are typically brought about by respiratory motion where, during a normal breathing cycle, abdominal organ movement has an average amplitude of 13 mm [22]. For larger rods of outer diameter greater than 8 mm, lower transmitter frequencies (1 kHz to 2 kHz) are necessary to ensure distortion-free tracking. This work identifies methods to reduce the tracking error brought about by the shielding effect of a metallic instrument. These are a reduction in the outer diameter of the instruments, the use of a suitable material with a lower electrical conductivity, or a reduction in the magnetic field frequency in exchange for lower update rates.

This work presents the first known evidence validating the use of electromagnetic sensors positioned within the shaft of metal instruments for tracking in clinical settings for applications such as laparoscopic surgery, robotics, arthroscopy and orthopaedics. As such, the results will be of considerable interest to users looking to use magnetic tracking in image-guided interventions where the sensor can now be positioned within the metal shaft itself within the design parameters outlined. This work has immediate applications in procedures such as minimally invasive cholecystectomy, where laparoscopic surgery has become the standard of care [23]. For these procedures, typical port diameters range from 2 mm to 12 mm.

## Figures and Tables

**Table 1:** Parameters of rods used for experimental verification.

Rod	Material	$r_{i}$ (mm)	$r_{o}$ (mm)	σ (MS/m)
1	316 L Stainless Steel	3.4	4	1.35
2	316 L Stainless Steel	5	6	1.33

**Table 2:** Tracking errors at different frequencies in the presence of no rod, rod 1 and rod 2.

	RMSE (mm)			ME (mm)			STD (mm)		
Frequency (kHz)	No Rod	Rod 1	Rod 2	No Rod	Rod 1	Rod 2	No Rod	Rod 1	Rod 2
1	0.89	1.09	1.09	0.71	0.89	0.92	0.53	0.63	0.59
5	0.98	1.16	1.55	0.79	0.94	1.29	0.58	0.68	0.85
10	1.26	1.69	4.55	1.01	1.38	3.97	0.75	0.98	2.21
15	1.38	2.75	10.01	1.13	2.30	8.83	0.80	1.52	4.72
20	2.33	12.74	25.53	1.89	11.08	22.17	1.37	6.30	12.68

## Data Availability

Data are contained within the article.

## Abbreviations

The following abbreviations are used in this manuscript:

EMT	Electromagnetic tracking
DAC	Digital-to-analog converter
ADC	Analog-to-digital converter
IA	Instrumentation amplifier
FFT	Fast Fourier transform
IR	Infrared
RMSE	Root-mean-square error
ME	Mean error
STD	Standard deviation
